# A Retrospective Study of Invasive Candidiasis in a Tertiary Teaching Hospital

**DOI:** 10.7759/cureus.96913

**Published:** 2025-11-15

**Authors:** Priti Dave, Ishani Mishra, Girik Bhatia

**Affiliations:** 1 Internal Medicine, Symbiosis University Hospital and Research Center (SUHRC) Symbiosis Medical College for Women (SMCW) Symbiosis International University (SIU) Lavale, Pune, IND; 2 Internal Medicine, Bharati Vidyapeeth Medical College, Pune, IND

**Keywords:** antifungal resistance, blood stream infection (bsi), candida auris, candida parapsilosis, candida tropicalis, invasive candidiasis, nosocomial infection, risk factors

## Abstract

Introduction

Invasive candidiasis is a leading cause of fungal infections in hospitalized and critically ill patients, with high morbidity and mortality. There are numerous factors which may predispose to its development. In recent years, there has been a change in the etiological spectrum, and emerging resistance to antifungal drugs seen in some patients, further complicating management.

Aims and objectives

The aim of this study was to analyze the epidemiological and microbiological profile of invasive candidiasis in a tertiary teaching hospital, with a focus on species distribution, antifungal resistance patterns, clinical risk factors, and patient outcomes.

Materials and methods

This retrospective study included all consecutive adult inpatients with invasive candidiasis admitted to Bharati Hospital & Research Centre, Pune, India, from April 2019 to March 2022. This cohort represents all eligible cases (n=86) identified during the study period, and therefore, the sample size was determined by case availability rather than a prior statistical calculation. Data were collected to evaluate species distribution and evolving epidemiology, identify associated risk factors, analyse antifungal susceptibility patterns, and determine mortality outcomes.

Results

*Candida albicans* (CA) was isolated in 19 (22.09%) out of 86 cases, while non-albicans Candida (NAC) in 67 (77.91%) of patients. The most common species isolated among NAC was* Candida parapsilosis* in 28 (32.56%) cases, followed by *Candida tropicalis* in 19 (22.09%) cases and *Candida auris* in 12 (13.95%) cases. Out of 86 patients with invasive candidiasis, we found antibiotic use in 81 (94.2%) cases, intravascular catheters (central venous line/hemodialysis catheter) in 65 (75.6%) cases, Foley catheter in 49 (57%) cases, diabetes mellitus (DM) in 41 (47.7%) cases, chronic kidney disease (CKD)/acute kidney injury (AKI) in 41 (47.7%) of patients, and surgery in 28 (32.6%) patients. Other associated factors found were steroid and immunosuppressive use, total parenteral nutrition, cancer chemotherapy, human immunodeficiency virus (HIV) positive status and prosthetic devices. Mortality was seen in 24 (27.9%) patients. Patients with two or more associated illnesses had mortality 2.36 times more than those with less than two illnesses (p value = 0.016).

Conclusion

Invasive candidiasis in hospitalized patients remains a significant challenge, driven by rising non-albicans species and increasing antifungal resistance. Early recognition in high-risk patients, antimicrobial stewardship, and meticulous catheter care are essential to prevent morbidity and mortality. Improved availability of more sensitive tests to detect invasive candidiasis infections is the need of the day so that antifungals can be started at the correct time.

## Introduction

Invasive candidiasis accounts for 70-90% of invasive fungal infections globally and is associated with significant morbidity and mortality [1]. While* Candida albicans* has historically been the predominant pathogen, recent surveillance indicates a rising burden of non-albicans Candida (NAC) species worldwide. This shift has been increasingly reported worldwide, where C. albicans once predominated but NAC species now contribute substantially to invasive disease [2]. In India, invasive candidiasis is reported to be the third most common cause of blood stream infections; its prevalence ranges from 6-18% in different parts [3]. Invasive candidiasis is a blood stream infection (candidemia) and/or deep-seated organ infection by the fungus *Candida*. There are at least 15 *Candida* species that affect humans, but not all are commonly seen in clinical practice. *Candida* is often present as a commensal prior to causing invasive candidiasis. Increased colonization or decreased host immunity play an important role in its genesis.

The clinical spectrum of invasive candidemia is variable with mortality as high as 60% [4]. Currently, blood culture is the diagnostic benchmark for detecting invasive candidiasis infection. However, the sensitivity of this test is 50% and it takes a prolonged incubation time to detect it, which results in infections going undetected. There are other non-culture diagnostic tests which help in detecting invasive candidiasis, like mannan antigen, anti-mannan antibody and *Candida albicans* germ tube antibody test (CAGTA), but they have not been widely validated and are not available easily [5]. This leads to the use of empirical antifungal agents which may increase the development of resistant* Candida *infections. This study was undertaken to know the changing etiological pattern, if any, and the antifungal sensitivity profile which will help us select the correct antifungal medications and decrease mortality in patients. We also tried to revisit the risk factors associated with patients of invasive candidiasis so as to understand the precautions that can be taken to reduce the incidence of the same.

## Materials and methods

This retrospective observational study was conducted at Bharati Hospital and Research Centre, Pune, India, and included all adult inpatients (≥18 years) diagnosed with invasive candidiasis between April 1, 2019, and March 31, 2022. Eligible patients were those with at least one isolation of *Candida* species from a normally sterile site, including blood, sterile body fluids, or deep-tissue specimens during the study period. Patients with only superficial *Candida* infections or with non-*Candida* fungal infections were excluded. Over the three-year period, 92 patients met these criteria; however, complete clinical records were available for only 86 individuals, who were therefore included in the final analysis. As this comprised all consecutive eligible cases with complete data, the sample size reflected case availability rather than a predetermined statistical calculation.

The study objectives were to identify and analyze the risk factors associated with invasive candidiasis, determine the species of *Candida* causing invasive candidiasis and their antifungal sensitivity patterns, and evaluate the clinical outcomes of patients in terms of survival and mortality.

Patient records were reviewed through hospital medical files and microbiology laboratory databases. Inpatient department (IPD) numbers of patients with invasive candidiasis were obtained from microbiology records. Demographic details, clinical presentation, and risk factors such as central venous line use, prolonged hospitalization, antibiotic exposure, diabetes mellitus (DM), HIV infection, cancer chemotherapy, immunosuppressive therapy, steroid use, history of organ transplant, recent surgery, kidney failure, total parenteral nutrition (TPN), hemodialysis, indwelling catheters, and prosthetic devices were documented. Laboratory and imaging data included complete blood count, blood glucose profile, renal and liver function tests, blood cultures, and chest radiography. Additional investigations were noted where clinically indicated, including urine culture for candiduria, echocardiography when endocarditis was suspected, and abdominal ultrasound or CT for identification of intra-abdominal sources. Antifungal therapy details (drug, dosage, and duration) and patient outcomes (survival vs. mortality) were recorded. All Ethics approval was obtained from the Institutional Ethics Committee (reference number: BVDUMC/IEC/20C).

Statistical analysis was carried out using SPSS Statistics version 28.0 (IBM Corp., Armonk, NY, USA). Categorical variables were expressed as number (N) and percentage. Relative risk ratios were calculated using 2x2 contingency tables to assess the association between risk factors and mortality. Statistical significance of associations was determined using Pearson Chi-Square test. A *p*-value <0.05 was considered statistically significant.

## Results

**Figure 1:**
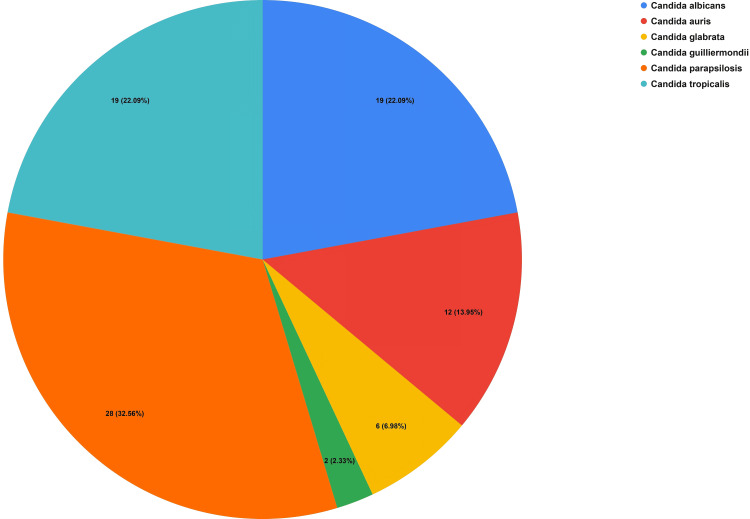
Percentage Distribution of Various Candida Species

During the three-year study period, a total of 86 patients met the inclusion criteria (mean age 51.99 years, range 21-80 years), of whom 58 (67.4%) were male and 28 (32.6%) female (Table 1). *Candida albicans* (CA) was identified in 19 (22.09%) patients, whereas non-albicans candida (NAC) species predominated in 67 (77.91%) cases. The most common NAC were *Candida parapsilosis* in 28 (32.56%), *C. tropicalis* in 19 (22.09%), and *C. auris* in 12 (13.95%) patients, followed by *C. glabrata* in 6 (6.98%) and* C. guilliermondii* in 2 (2.33%) patients (Figure 1).

**Table 1:** Demographic, Clinical, and Outcome Characteristics of Patients with Invasive Candidiasis (N=86) CLD – Chronic liver disease; UTI – Urinary tract infection; CKD – Chronic kidney disease; AKI – Acute kidney injury; TPN – Total parenteral nutrition; HIV – Human immunodeficiency virus.

Characteristic	N (%) or Mean (Range)
Demographics	
Age, years (mean, range)	51.99 (21–80)
Male sex	58 (67.4%)
Female sex	28 (32.6%)
Comorbidities	
Cardiac disease	39 (45.3%)
Sepsis (Bacterial)	21 (24.4%)
Sepsis with shock (Bacterial)	7 (8.1%)
Respiratory disease	15 (17.4%)
Chronic liver disease (CLD)	7 (8.1%)
Urinary tract infection (UTI)	3 (3.5%)
Risk Factors	
Antibiotic use	81 (94.2%)
Intravascular catheter	65 (75.6%)
Foley catheter	49 (57.0%)
Diabetes mellitus	41 (47.7%)
CKD/AKI	41 (47.7%)
Surgery	28 (32.6%)
Steroid or immunosuppressive therapy	24 (27.9%)
Total parenteral nutrition (TPN)	8 (9.3%)
Cancer chemotherapy	6 (7.0%)
Prosthetic device	2 (2.3%)
Human immunodeficiency virus (HIV) infection	2 (2.3%)
Outcomes	
Mortality	24 (27.9%)
Survival	62 (72.1%)

Prior antibiotic exposure was seen in 81 out of 86 (94.2%) patients, intravascular catheters in 65 (75.6%), and Foley catheters in 49 (57.0%) patients. Other common risk factors included diabetes mellitus (DM) and chronic kidney disease (CKD)/acute kidney injury (AKI) in 41 patients each (47.7% each), and recent surgery in 28 (32.6%) patients. Additional comorbidities included cardiac disease in 39 (45.3%), bacterial sepsis in 21 (24.4%), respiratory disease in 15 (17.4%), sepsis with shock and chronic liver disease (CLD) in seven cases each (each 8.1%) (Table 1).

**Figure 2:**
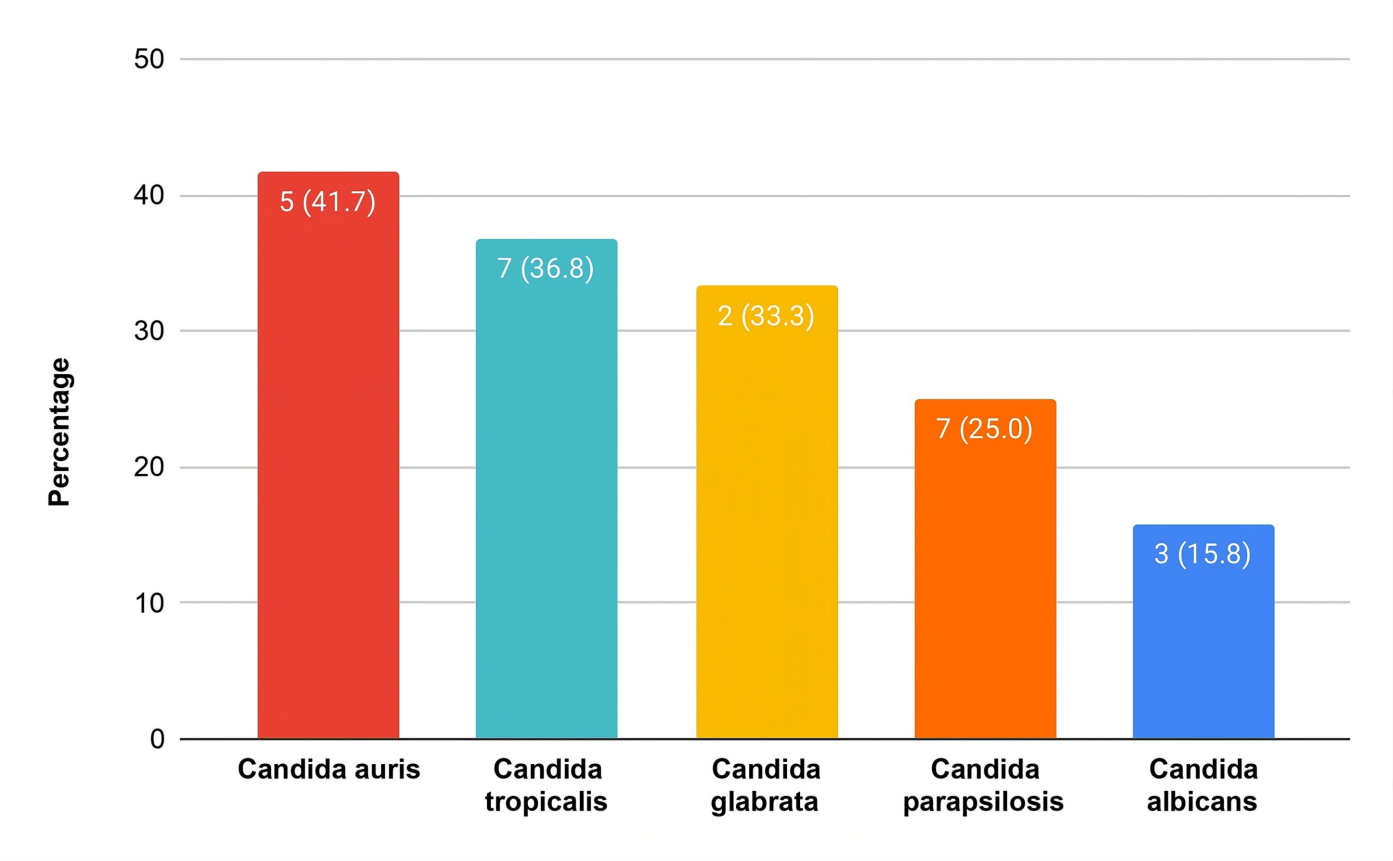
Mortality in Different Candida Species

Overall mortality was 27.9% (24/86) (Table 1). Patients with ≥2 comorbid illnesses had significantly higher mortality than those with <2 (relative risk [RR] = 2.36, *p*=0.016*). Higher risk trends were seen with ≥3 risk factors and >4 total factors, although not statistically significant (Table 2). Notably, *Candida auris* infection carried especially high mortality of 41.7% (5/12) (Figure 2).

**Table 2:** Relative Risk Ratio of Mortality Note: Mortality values represent the number of deaths (n), total patients (N), and percentage mortality in each group. Relative risk (RR) is shown for comparison groups, with reference groups set at 1.0. *P*-values were calculated using Pearson Chi-Square test; *p* < 0.05 was considered statistically significant. Statistically significant *p*-values are marked with an asterisk (*). RR = Relative Risk; CI = Confidence Interval; n = number of deaths; N = total patients; % = percentage; *p*-value = probability value.

No. of Risk Factors	Mortality (n/N, %)	Relative Risk (RR)	Lower 95% CI	Upper 95% CI	Chi-square	*P*-value
>3	20/60 (33.3%)	2.16	0.821	5.715	2.905	0.088
≤3	4/26 (15.4%)	1.00	—	—	—	—
No. of Associated illness						
>2	8/15 (53.3%)	2.36	1.247	4.490	5.838	0.016*
≤2	16/71 (22.5%)	1.00	—	—	—	—
Total (No. of Risk factors + Associated illness)						
>4	21/63 (33.3%)	2.55	0.840	7.766	3.448	0.063
≤4	3/23 (13.0%)	1.00	—	—	—	—

**Figure 3:**
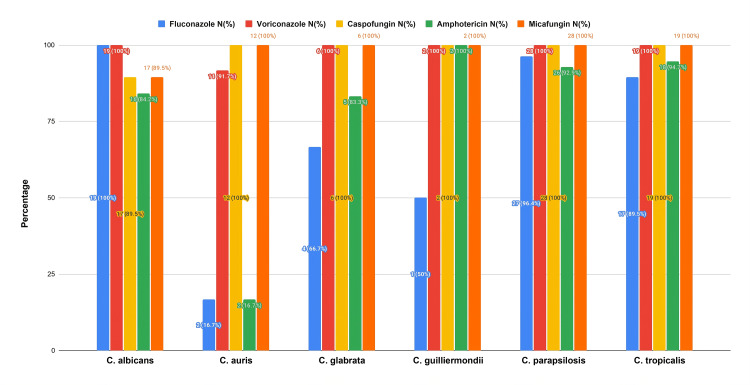
Antifungal Susceptibility Patterns of Candida Species

We observed fluconazole resistance in 16 (18.6%) and amphotericin resistance in 17 (19.8%) isolates. Resistance was heavily skewed toward *C. auris* (10/12, i.e., 83.3% fluconazole and amphotericin resistance). Resistance to fluconazole was also noted in two out of six *Candida glabrata* (33.3%),* *two out of 19 *C. tropicalis* (10.5%) and one out of 28 *C.* *parapsilosis *(3.6%) isolates. All* C. albicans *isolates were susceptible to fluconazole and voriconazole (Figure 3).

## Discussion

In this tertiary-care study of 86 invasive candidiasis cases, we found a marked predominance of non-albicans species. Over three-quarters of isolates were NAC, led by *C. parapsilosis*, *C. tropicalis*, and *C. auris*. This was consistent with recent data from 2024-2025 demonstrating that invasive candidiasis is no longer dominated by *Candida albicans* as non-albican species were more commonly isolated [6-7]. This significant change of pattern from CA to NAC is a concern for clinicians as it is important to ponder before giving antifungals empirically when a patient's fever doesn’t settle with even higher antibiotics. However, this may depend upon the patient's age, geographical location and previous antifungals used [3]. While several Indian studies identify *C. tropicalis* as the most frequent isolate, accounting for 35-45% of cases [3,8,9], *C. albicans* continues to predominate in some Western regions, including the United States [10]. These findings highlight regional variability in species distribution and reinforce the importance of local epidemiological surveillance to guide antifungal therapy. *Candida parapsilosis* is recognized as one of the most frequently isolated non-albicans species in invasive candidiasis [7]. Although traditionally considered less virulent than *C. albicans*, it often demonstrates reduced susceptibility to azoles and echinocandins. Unlike *C. albicans, C. parapsilosis* does not always require prior colonization and is notable for its potential for horizontal transmission in intensive care settings. Its ability to form biofilms on intravascular and other indwelling medical devices further emphasises the need for strict infection-control and catheter-care practices [11].

In our study, prior antibiotic use was the most prominent risk factor for invasive candidiasis. This high prevalence of previous antibiotic use has been found in similar studies which have shown that antibiotic exposure disrupts gut microbiota and gut immunity. It also alters the gut barrier. Antibiotics with high gut concentrations and strong anaerobic activity appear particularly associated with candidemia [12,13]. Candidemia is also associated with gut surgeries, especially anastomosis leakage and is also seen with necrotizing pancreatitis [12]. In addition, device-related factors such as central venous and hemodialysis catheters, Foley catheters, and prosthetic devices were frequently observed in our study. Indwelling devices can sometimes be kept a little longer than desired, sterilized and reused due to patients' financial constraints, and handled excessively. This may contribute to the development of blood stream infections.

We noted a mortality of 27.9% in our study group. One study reported a high crude mortality rate of 25-50% [14]. Xiao et al. recorded a high all-cause mortality rate of 51.2% [15]. These figures highlight the persistently high mortality associated with invasive candidiasis despite advances in diagnostics and therapy. Management requires a minimum of 14 days of antifungal treatment after the first negative blood culture, which prolongs hospitalization and adds to financial costs, risk of nosocomial infections, and drug-related adverse effects.

Antifungal resistance was an important finding in this study. We observed noteworthy resistance to both fluconazole and amphotericin, particularly among NAC isolates. *C. albicans* remained fully susceptible to fluconazole, which is consistent with recent surveillance reports [6-7]. Fluconazole resistance has been linked to ERG11 gene mutations and is more commonly reported in species such as *C. krusei, C. glabrata*, and some other rare species [12,16]. Among NAC species, *C. auris *demonstrated the most pronounced multidrug-resistant profile, consistent with findings from other studies [17,18]. Reduced susceptibility in *C. glabrata, C. tropicalis,* and *C. parapsilosis* was also observed, although at lower levels. Hence, with the changing spectrum from *Candida albicans* to non-albicans species, heightened vigilance and antifungal stewardship are essential to limit the emergence and spread of resistance. Mortality among patients with *Candida auris* infection was particularly striking, with 41.7% of affected patients in our cohort dying, comparable to the 52.5% fatality rate reported in a retrospective study conducted in Oman [19]. These figures highlight the persistently high mortality associated with multidrug-resistant species such as *C. auris.*

Limitations

The limitations of this study include its retrospective design and modest sample size. The absence of a control group restricted direct comparisons. In addition, the analysis was descriptive and did not adjust for all potential confounders. These factors should be considered when interpreting the findings.

## Conclusions

Invasive candidiasis remains a serious threat in critically ill hospitalized patients, with a considerable mortality burden. A clear shift toward non-albicans species, coupled with emerging antifungal resistance, particularly with the high mortality seen in *Candida auris*, underscores the evolving epidemiology of this infection. Judicious use of broad-spectrum antibiotics is essential to reduce the risk of invasive candidiasis, as prolonged antibiotic exposure remains a major contributor. Similarly, meticulous aseptic handling and timely removal of intravascular catheters are critical, given their role as an important and preventable risk factor. Strengthening antimicrobial stewardship, device-care practices, and early diagnostic capabilities will be key to improving patient outcomes.
